# Editorial: Neuroimaging of non-motor deficits in movement disorders

**DOI:** 10.3389/fnhum.2023.1203527

**Published:** 2023-05-09

**Authors:** Edward Ofori, Alex Wiesman, Xiongfei Wang, Max Kurz

**Affiliations:** ^1^Pathomechanics & Neuroimaging Lab, College of Health Solutions, Arizona State University, Phoenix, AZ, United States; ^2^Montreal Neurological Institute, McGill University, Montreal, QC, Canada; ^3^Sanbo Brain Hospital, Capital Medical University, Beijing, China; ^4^Institute for Human Neuroscience, Boys Town National Research Hospital, Omaha, NE, United States

**Keywords:** Parkinson's disease, movement disorder, non-motor deficits, cognition, neuroimaging, parkinsonism, machine learning

## Introduction

Parkinson's disease (PD) is a complex neurodegenerative disorder that manifests not only in motor symptoms but also in non-motor features such as cognitive impairments and neuropsychiatric issues. The heterogeneity of PD and other movement disorders has long posed challenges in diagnosis, prognosis, and treatment. Recently, advances in neuroimaging and machine learning have provided new insights into the pathophysiology of PD and its subtypes, paving the way for personalized therapeutic approaches. In this editorial, we discuss four recent studies that have utilized these cutting-edge techniques to unravel the complexities of motor and non-motor features in movement disorders.

## Identifying PD subtypes through multimodal MRI indices

Cao et al. employed hierarchical clustering analysis based on multimodal MRI indices, amplitude of low-frequency fluctuation (ALFF), and gray matter volume (GMV) to identify two distinct PD subtypes with unique neurodegenerative patterns. The “diffuse malignant subtype” exhibited reduced ALFF in the visual-related cortex and extensive GMV reduction, coupled with severe motor and cognitive impairments. In contrast, the “mild subtype” displayed increased ALFF in the frontal lobe, temporal lobe, and sensorimotor cortex, and a slight decrease in GMV, with milder motor and cognitive impairments. Cao et al. highlight the potential of unsupervised cluster analysis based on multimodal MRI indices to inform accurate prognosis and personalized treatment plans for PD patients.

## STN-DBS treatment for PD patients with treatment-resistant depression

Zhang et al. investigated the efficacy of subthalamic nucleus deep brain stimulation (STN-DBS) in treating both motor and depressive symptoms in PD patients with treatment-resistant depression (PD-TRD). The results indicated that STN-DBS significantly improved motor and depressive symptoms, reduced the need for antiparkinsonian and antidepressant medications, and enhanced daily living activities and health-related quality of life after 1 year of treatment. Moreover, the study revealed widespread abnormalities in cerebral regional glucose metabolism in PD-TRD patients, which were partially recovered following STN-DBS treatment. These findings support the potential of STN-DBS as a promising treatment option for managing motor and depressive symptoms in PD-TRD patients.

## Decreased prefrontal glutamate levels and cognitive impairment in PD

Buard et al. examined the relationship between decreased prefrontal glutamate levels and cognitive impairment in PD patients using proton magnetic resonance spectroscopy. The researchers observed significant differences in prefrontal glutamate levels between PD patients with normal cognition and those with dementia. These findings suggest that fluctuations in prefrontal glutamate may serve as a biomarker for the progression of cognitive impairments in PD patients, warranting further investigation through larger magnetic resonance spectroscopy studies.

## Identifying depressed ET patients using machine learning and neuroimaging

Li et al. aimed to identify patients with depressed essential tremor (ET) by utilizing machine learning multivariate pattern analysis (MVPA) combined with global brain connectivity (GBC) mapping from resting-state fMRI. The study successfully distinguished depressed ET patient participants from those with non-depressed ET, primary depression, and healthy controls (HCs) using multiclass Gaussian process classification (GPC) and binary support vector machine (SVM) algorithms. The significant discriminative features were mainly located in cerebellar-motor-prefrontal cortex circuits, with GBC changes in these circuits correlating with clinical depression severity in patients with depressed ET. The Li group demonstrate the potential of combining machine learning and neuroimaging to identify patients with comorbid depressed ET and shed light on the network pathogenesis underlying depression in these patients.

## Final thoughts

The studies discussed in this editorial highlight the powerful potential of neuroimaging and machine learning techniques in disentangling the heterogeneity of motor and non-motor features of Parkinson's disease and essential tremor. These state-of-the-art approaches have shown success in identifying clinically relevant subtypes, differentiating patients with specific comorbidities from those without, and uncovering the neural mechanisms underlying these conditions. As our understanding of movement disorders continues to evolve, these findings will undoubtedly inform more accurate diagnoses, refined prognoses, and personalized treatment plans, ultimately improving the quality of life for patients living with these complex disorders.

## Author contributions

All authors listed have made a substantial, direct, and intellectual contribution to the work and approved it for publication.

